# High-definition brain stimulation targeting separate regions leads to differential word retrieval outcomes in patients with primary progressive aphasia: a pilot study

**DOI:** 10.3389/fneur.2025.1630103

**Published:** 2025-09-17

**Authors:** Christine Sofka Dugas, Hsueh-Sheng Chiang, Paulina Devora, Katelyn Lucas-Mendoza, Christine Abasi, Ashna Adhikari, Trung Nguyen, Alexander Frolov, Brendan J. Kelley, Christian LoBue, Raksha A. Mudar, John Hart

**Affiliations:** ^1^Department of Behavioral and Brain Sciences, The University of Texas at Dallas, Richardson, TX, United States; ^2^Department of Neurology, Berenson-Allen Center for Noninvasive Brain Stimulation, Beth Israel Deaconess Medical Center, Boston, MA, United States; ^3^Department of Neurology, University of Texas Southwestern Medical Center, Dallas, TX, United States; ^4^Department of Psychiatry, University of Texas Southwestern Medical Center, Dallas, TX, United States; ^5^Department of Neurological Surgery, University of Texas Southwestern Medical Center, Dallas, TX, United States; ^6^Department of Speech and Hearing Science, University of Illinois Urbana-Champaign, Champaign, IL, United States

**Keywords:** primary progressive aphasia, apraxia of speech, word retrieval deficits, transcranial direct current simulation, pre-SMA stimulation, LIFG stimulation, EEG

## Abstract

**Background:**

Word retrieval deficits are the most prominent symptoms reported in primary progressive aphasia (PPA) and related syndromes. Current treatments, such as speech and language therapy, have shown limited success, highlighting the need for alternative non-pharmacological interventions with high-definition transcranial direct current stimulation (HD-tDCS) emerging as a promising tool.

**Objective:**

This study aimed to evaluate the effects of HD-tDCS on word retrieval function in individuals with PPA by comparing two stimulation sites: the pre-supplementary motor area (pre-SMA) and the left inferior frontal gyrus (LIFG), and to assess the relative benefits of each site.

**Methods:**

Eight individuals with PPA underwent 10 sessions of open-label HD-tDCS targeting either the LIFG (*n* = 4) or pre-SMA (*n* = 4). Word retrieval was assessed at baseline, immediately post-stimulation, and at 8-week follow-up. Electrophysiological measures, including event-related potentials during a non-verbal Go-NoGo task, were also collected to explore neural mechanisms.

**Results:**

LIFG stimulation yielded statistically significant improvements in phonemic fluency at immediate post testing compared to baseline, with 25–50% showing clinically meaningful improvement. Clinically meaningful improvement was observed in category fluency in 25–50% of the patients receiving stimulation at either site. Lastly, electrophysiological measures indicated HD-tDCS targeting LIFG differentially modulated event-related potential effects during non-verbal Go-NoGo tasks.

**Conclusion:**

This research provides preliminary evidence supporting the use of both traditional (LIFG) and alternative (pre-SMA) stimulation sites for treating word retrieval deficits in individuals with PPA. The findings also suggest potential neural mechanisms of HD-tDCS intervention, which can inform future designs of non-invasive brain stimulation for cognitive symptoms in PPA.

**Clinical trial registration:**

Clinicaltrials.gov, identifier NCT05368350.

## Introduction

Primary progressive aphasia (PPA) encompasses a cluster of clinical syndromes associated with progressive deficits involving speech and language functions in earlier stages, followed by deficits in other cognitive functions in later stages, typically due to an underlying neurodegenerative disease (frontotemporal lobar degeneration, corticobasal degeneration, progressive supranuclear palsy, Alzheimer’s disease) (1, 2). Three primary phenotypes have been described (3): nonfluent/agrammatic variant PPA (nfvPPA) presents with telegraphic speech/agrammatism associated with atrophy in the left posterior fronto-insular region; semantic variant PPA (svPPA) presents with impaired naming abilities and degraded semantic knowledge associated with atrophy in the anterior temporal lobes; logopenic variant PPA (lvPPA) presents with impaired sentence repetition and word finding difficulty associated with atrophy in the left posterior perisylvian and parietal regions of the brain. Additionally, primary progressive apraxia of speech (PPAOS) is a motor speech impairment that may present as a distinct syndrome from PPA, which is associated with atrophy and hypometabolism in the superior mesial prefrontal cortex (including the pre-supplementary motor area) and may co-occur with nfvPPA (4, 5). In general, impaired word retrieval function is one of the most common deficits across all PPA types (1, 3).

The general treatment for PPA-related word retrieval deficits has been speech-language therapy (SLT), tailored to strengthen semantic, phonological, or fluency aspects of language, with variable to limited success (6, 7). While SLT has been shown to be beneficial in improving language treatment outcomes in PPA (8), this type of intervention requires long-term adherence in order to maintain benefits given the progressive and degenerative nature of PPA (9). However, adherence to treatment has been shown to decline as time goes on (6, 10). SLT research has also noted not all individuals with PPA respond to treatment such that treatment responses are variable and depend on factors, including patient motivation, severity of deficits, and stage of disease progression (11). Despite these limitations, SLT remains the primary intervention for word retrieval deficits in PPA, with no other validated treatment options currently available.

Recent advances in non-invasive brain stimulation have provided novel therapeutic opportunities for remediating language and speech difficulties in neurologic patients including PPA (12–15). Non-invasive brain stimulation methods such as transcranial direct current stimulation (tDCS) or repetitive transcranial magnetic stimulation (rTMS) may selectively modulate activity in the brain regions that can be affected by PPA, resulting in preservation or even improvement of the targeted functions (14–16). We focused on tDCS, specifically a refined technique called high-definition tDCS (HD-tDCS), given its safety, tolerability, feasibility and efficacy profiles (17). Although exact mechanisms are still being investigated, it is thought that tDCS transiently depolarizes (via anodal stimulation with influx of electric current) or hyperpolarizes (via cathodal stimulation with efflux of electric current) neuronal resting membrane potentials, affecting the rate of neuronal firing and modulating cortical excitability (18, 19). Multiple and/or prolonged sessions can induce long-term potentiation of neurons in the targeted region, as well as even more widespread change in the connectivity across a neural circuit (20).

Coemans et al. (12) systematically reviewed the literature and suggested that tDCS is a generally effective tool to improve language outcomes in PPA. Early intervention should be given consideration since the presence of intact neural circuitry may enhance the benefit from neuromodulation (6, 7). Nissim et al. (13) conducted a separate meta-analysis/review and found that there was no clear conclusion of neuromodulation efficacy due to the variability between selected studies, including which PPA subtype, variability in behavioral treatment approaches, and variability in stimulation protocols and outcome measures. Similarly, a recent Cochrane review concluded that there is little or unclear evidence of the benefits of non-invasive brain stimulation in treating word retrieval deficits in PPA, given that most of the studies so far have not been well randomized and sham-controlled, and the few randomized controlled studies are insufficient for formal meta-analysis (14). The Cochrane review emphasized the need to optimize protocol and noted that there is a general lack of attention related to cognitive and functional outcomes other than language measures that may also impact overall function and quality of life in PPA patients. Non-invasive brain stimulation is often studied in combination with SLT or other computerized cognitive tasks, other than a few exceptions (14, 21–23). Therefore, little is known of the benefits of non-invasive brain stimulation in treating word retrieval deficits in PPA beyond the effects of speech-language therapy alone.

The brain region(s) that should be targeted to improve word retrieval in PPA is still unclear (14, 15). Coemans et al. (12) suggest that the optimal location for tDCS stimulation is over regions shown to be affected by lesions or engaged by the targeted language functions as opposed to regions that may contribute to compensatory strategies. In view of that, the the left inferior frontal gyrus (LIFG), associated with word retrieval function and speech production, appears to be an excellent candidate and has shown promise in remediating word retrieval deficits across different PPA types with or without apraxia of speech (12, 24, 25). A neural circuit central to word retrieval function put forth by our group involves the pre-supplementary motor area (pre-SMA) in addition to the LIFG, and a fronto-caudate-thalamic circuit (26, 27). The pre-SMA is proposed to play a central role in domain-general function involving speech production and fluency, while the LIFG may be more specific to lexico-semantic selection and retrieval (27). Hence atrophy or dysfunction in either pre-SMA or LIFG, and often both, can be associated with reduced word retrieval in PPA, including reduced verbal fluency, agrammatism, and impaired motor speech function (1, 4, 5). We therefore aimed to examine the differential effects of HD-tDCS targeting either the pre-SMA or the LIFG in PPA. Although there is no prior report of pre-SMA modulation in treating PPA, several studies have shown promise in targeting the pre-SMA to improve retrieval of verbal information in healthy adults and patient populations (28–31). Studies have also shown post-treatment changes in the frontal neural responses following pre-SMA stimulation (27, 31). Previous studies have compared different stimulation montages in PPA, specifically comparing the left dorsolateral prefrontal cortex (DLPFC) to left inferior parietal lobule (IPL), (32), or the LIFG to left IPL (33, 34). Despite the role the pre-SMA plays in word retrieval, stimulation to this area has yet to be studied or compared to other montages (e.g., LIFG) in PPA.

In addition to measuring behavioral effects, we also sought to investigate changes in neural activity (i.e., neuroplasticity of the neural circuit involved) due to HD-tDCS intervention using electroencephalography (EEG), a direct measure of neuronal activity that may provide mechanistic evidence of tDCS effects (29–31). No study based on PPA thus far has examined EEG changes associated with tDCS. We focused on EEG activity during two Go-NoGo tasks with different levels of perceptual and semantic complexity. These tasks consistently elicit frontal event-related potential (ERP) components, as early as around 150–250 ms (N2/N200) and 300–500 ms (P3/P300) post-stimulus onset, indexing selection of the correct target object and inhibition of incorrect target objects thought to be generated by the underlying neural circuit involving the frontal regions (35–38). These ERP components have been linked to word retrieval deficits and thought to reflect cognitive control (and more domain general) aspects of word retrieval (28, 39), hence having the potential to serve as neural markers of intervention effects on word retrieval function.

We designed our study to achieve several goals. First, we tested the feasibility and tolerability of an open-label active HD-tDCS study using two separate target engagement sites, that is, the pre-SMA which is a novel target compared to the LIFG which is a more established target. We did not combine tDCS with speech/language treatment to isolate effects of stimulation and minimize variability between participants. Second, we focused on word/verbal production as the outcome measures, but also included other cognitive and language as supplemental measures to better capture potential tDCS effects that may also benefit PPA patients outside the language domain (14). Third, we aimed not only to report statistical significance, but also to report potential efficacy and clinically meaningful change, which is generally lacking in the literature (14, 15). Fourth, we analyzed EEG responses during the Go-NoGo tasks to measure changes in neuronal activity induced by cumulative tDCS treatment effects. In summary, we examined the effects of ten 20-min sessions of active anodal HD-tDCS targeting the pre-SMA compared to the LIFG on word retrieval function. We hypothesized that there would be differential effects comparing pre-SMA and LIFG stimulations. Specifically, pre-SMA stimulation would lead to changes in language (primary outcomes) as well as other cognitive domains (secondary outcomes), as the pre-SMA is thought to be more domain-general. In contrast, LIFG stimulation would lead to changes more specific to language function. We also hypothesized that changes in neural activity as measured by ERP would be more evident with the pre-SMA stimulation as opposed to LIFG stimulation, given that pre-SMA is thought to be one of the neural generators of these frontal ERP activities underlying response selection and inhibition during Go-NoGo tasks. Baseline ERPs of PPA patients were also compared to matched controls to better characterize such potential changes.

## Materials and methods

### Participants

Participants were right-handed native English-speakers either self-referred (through the Clinicaltrials.gov registry: NCT05368350) or referred from the memory-specialty clinic at UT Southwestern Medical Center. Participant selection was limited to individuals in the early stages of PPA, defined as a confirmed diagnosis within the past 3 years. All participants had an initial diagnosis of PPA or PPAOS made by a neurologist, which were confirmed or further specified following comprehensive speech and language assessment. Individuals who were no longer verbally communicative were excluded at the time of initial inquiry. Functional status was assessed using the Reisberg Functional Assessment Staging Scale (FAST) (40) and those with >4 on the scale were excluded. Hence clinically these patients were at the stage of mild cognitive impairment or mild dementia, reflecting early-stage PPA. Participants who met the initial inclusion criteria (*n* = 11) were invited to have a first visit and completed baseline testing. Three were excluded from the data analysis: one (svPPA) was found to be more impaired than previously stated and was unable to complete baseline testing, one (nfvPPA) was excluded as English was a second language, and one (lvPPA) withdrew due to reduced tolerance of participation schedule (i.e., change in routine) and reported subjective decline in cognition after completing eight stimulation sessions. Eight PPA patients (*M*_age_ = 74.1 (range 69–79), female = 2) completed intervention and were included in the overall analysis. All participants who completed intervention provided relevant medical history, which is summarized in Supplementary material. Although EEG data from all eight participants were included in the baseline analysis, post-intervention EEG data were unavailable for PPA08 due to technical issues, resulting in seven participants with complete pre-post data.

An age- and sex-matched group of cognitively normal subjects (*n* = 7, *M*_age_ = 71.0 [range 67–79], female = 2) collected from prior studies [subgroup in Chiang et al. (41)] who underwent the identical EEG tasks were also included for EEG analysis to establish baseline ERP differences in PPA (PPA08 included for this baseline analysis) compared to this normal control group.

### Baseline measures and PPA classifications

Baseline assessments included the Raven’s Progressive Colored Matrices (RPCM) (42) to evaluate nonverbal reasoning and the Western Aphasia Battery–Revised (WAB-R) (43) to assess aphasia presence and severity using Aphasia Quotient (AQ) and Language Quotient (LQ) scores. AQ scores ranged from no aphasia (AQ > 93.8) to moderate aphasia (AQ = 51–75).

Motor speech impairments, including apraxia of speech (AOS) and dysarthria, were rated using tasks from the Apraxia Battery for Adults–Second Edition (ABA-2) (44) which included repetition, speech rate tasks, oral reading, an apraxia checklist, and a narrative sample. Two trained graduate clinicians (authors KLM and CA) completed the initial ratings, which were verified by a licensed speech-language pathologist (co-first author CSD), with final consensus reached among all three raters.

Three participants were classified with nfvPPA, three with the lvPPA, and two with progressive AOS or dysarthria and minimal aphasia. Four participants had comorbid AOS or dysarthria, while two exhibited motor speech deficits with little to no aphasia. AOS was further classified as either “prosodic,” characterized by slow speech rate and reduced pitch variation, or “phonetic,” involving inconsistent phonological and articulatory errors (45). Dysarthria was categorized as either hypokinetic, marked by reduced vocal intensity, limited breath support, and increased speech rate, or spastic, characterized by strained vocal quality and difficulty initiating speech (46–48). Progressive speech apraxia may present with dysarthria in parkinsonism-plus syndrome that can eventually progress to clinical phenotypes of progressive supranuclear palsy or cortico-basal syndrome, indicating subcortical involvement (49). However, we did not have evidence due to lack of neuropathology or other ancillary testing in the research study. PPA subtype representation and associated motor speech impairments were relatively balanced across stimulation groups (see Table 1). Participant demographics, diagnostic classification, and baseline measures are provided in Table 1.

**Table 1:** Participant demographic and baseline summary.

Patient ID	PPA01	PPA02	PPA03	PPA05	PPA07	PPA08	PPA09	PPA11
Demographic								
Age	79	74	69	72	74	68	75	76
Sex	Male	Male	Female	Male	Male	Female	Male	Female
Education (yrs)	14	14	12	14	18	16	16	16
Handedness	Right	Right	Right	Right	Right	Right	Right	Right
Race	C	C	C	C	C	C	C	C
Ethnicity	NH	NH	NH	NH	NH	NH	NH	NH
Stimulation type	PreSMA	PreSMA	PreSMA	LIFG	LIFG	LIFG	PreSMA	LIFG
Classification	nfvPPA/AOS	lvPPA	lvPPA	nfvPPA	PAOS	nfvPPA/AOS	PAOS^a^	lvPPA
Symptom onset (yrs)	3	4	6	4	3	2	2	6
Years since diagnosis	1	1	3	3	1	1	1	1
Language								
WAB-R								
Spont speech (20)	14	14	17	13	19	12	18	15
Comprehension (10)	9	8.05	5.85	9.6	10	20	9.85	9.9
Repetition (10)	8.4	5.2	8.6	8.6	10	8.6	9.6	8.4
Naming (10)	9.6	7.9	6.4	9.8	9.3	10	8.6	8.2
WAB AQ (100)	82	70.3	75.7	82	96.6	79.4	92.1	83
WAB LQ (100)	87.2	75.4	75.5	84.4	95.1	89.8	92.45	83
AQS (0–4)	1	2	2	1	0	1	1	1
Motor speech								
ABA-2 subtest 6	7	4	3	6	6	11	2	4
Apraxia SR (0–4)	2	0	0	1	1	3	0	0
Apraxia type	Prosodic	N/A	N/A	Mixed	Prosodic	Phonetic	N/A	N/A
Dysarthria SR (0–4)	2	0	0	1	1	1	2	2
Dysarthria type	Spastic	N/A	N/A	Spastic	Spastic	Hypokinetic	Spastic	Hypokinetic
Global intelligence								
RPCM %ile (Carolien)	99	100	27	64	100	15^b^	34	5
Functional								
FAST	2	2	4	4	3	2	4	6

^a^PPA09 initially diagnosed with PAOS, later baseline testing motor speech deficits better characterized by spastic dysarthria. ^b^Baseline RPCM score inconsistent with other baseline cognitive measures for PPA08. RPCM was reassessed at 8-weeks and scored in 94th percentile. Aphasia quotient severity (AQS) and Apraxia and dysarthria severity rating (SR) are scaled as follows: 0 = not present, 1 = mild, 2 = moderate, 3 = severe, 4 = profound. Dysarthria and apraxia severity ratings and type are informed by Duffy et al. (46) and Utianski et al. (73).

### Study design

This open-label study, pre-registered at the Clinicaltrials.gov website (NCT05368350), aimed to include patients with a diagnosis of primary progressive aphasia and/or primary progressive apraxia of speech. All primary and secondary outcome measures were administered at baseline, immediate post, and 8-weeks post HD-tDCS intervention and alternate forms were administered when available. Participants were pseudo-randomized into stimulation groups to balance for PPA subtypes and severity. Participant group assignment is summarized in Table 1. Informed consent was obtained from all participants (and their legal representatives) in accordance with Declaration of Helsinki and the protocols approved by the Institutional Review Boards of The University of Texas at Dallas (for PPA and normal control) and The University of Texas Southwestern Medical Center (for normal control).

### HD-tDCS protocol

**Figure 1:**
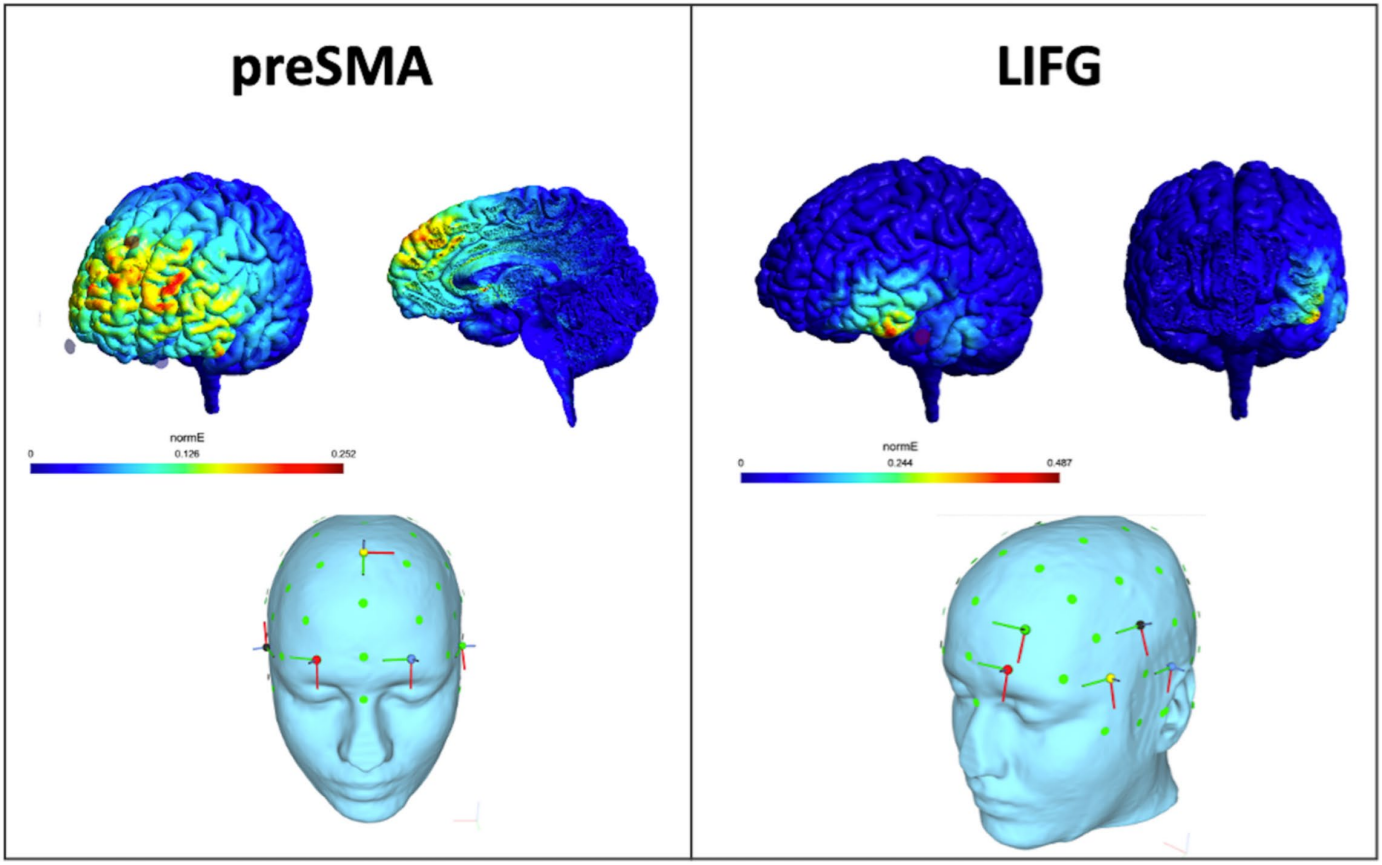
HD-tDCS montages and simulated electric field. These are HDtDCS montages used for the study based on a healthy sample subject using SimNIBS (72), with pre-SMA montage on the left (1 mA at Fz, −0.25 mA at FP1, FP2, F7, F8) and LIFG montage on the right (1 mA anode at F7, −0.25 mA at T7, FP1, AF3, FC5).

The HD-tDCS intervention protocol included one 20-min session per day over 10 days (five consecutive daily sessions per week for 2 weeks), using a Neuroelectrics Starstim system. During HD-tDCS, participants were instructed to sit quietly awake and at rest with their eyes open. The HD-tDCS montage comprised 5 Ag/AgCl 12-mm sintered pellet electrodes filled with conductive gel for the pre-SMA (one anode 1 mA at Fz and four 0.25 mA cathodes at FP1, FP2, F7, F8) and for the LIFG (one 1 mA anode at F7 and four 0.25 mA cathodes at T7, FP1, AF3, FC5 at 0.25 mA) (Figure 1). Electrodes were positioned in a neoprene cap. All patients underwent open-label active stimulation and were assigned to either the pre-SMA or the LIFG condition. Each stimulation session was preceded by a 30 s ramp up period to 1 mA current intensity followed by a 30 s ramp down period at the end. During stimulation sessions, a brief questionnaire was administered regarding adverse reactions intermittently (3–5×/10 sessions).

### Primary outcome measures

We use the term *word retrieval* to refer to the process of accessing and producing words belonging to any grammatical class. Word retrieval deficits are not limited to difficulties with nouns and verbs (i.e., content words), which are often observed in individuals with lvPPA and svPPA, but also extend to function words such as articles, prepositions, and conjunctions, which are more commonly affected in agrammatism associated with nfvPPA (1, 3, 50). Therefore, in this study, we selected tasks that tap into multiple aspects of word retrieval, including picture naming, verbal fluency, and discourse production, to serve as primary outcome measures.

The following standardized language assessments served as primary outcome measures administered at baseline, immediate post, and 8-weeks post stimulation: The Controlled Oral Word Association Test (COWAT) using “FAS” to assess phonemic fluency, the Category fluency tasks using “animals” (51), and the Boston Naming Test (BNT-60 items) (52). Discourse analysis was conducted on connected speech samples elicited through picture description using the Cookie Theft scene from the Boston Diagnostic Aphasia Examination (BDAE) (53). Samples were analyzed for words per minute (WPM) and two discourse measures: main concepts (MC) and core lexicon (CoreLex) targets (54–56). Scoring was performed by graduate student clinicians (authors: KLM, CA) and verified by a licensed speech-language pathologist (co-first author: CSD). Discourse analysis methods and secondary outcome measures and analysis are provided in Supplemental material.

### EEG task, EEG processing and ERP measurement

Word retrieval has been shown to engage semantic inhibition/selection mechanisms, which plays a critical role in lexicosemantic search and retrieval processes (26, 27). To examine these semantic inhibition and selection process in individuals with word retrieval impairments, we employed a Go/NoGo EEG task which engages a retrieval circuit implicated these retrieval processes (26, 27, 36, 39, 41).

**Figure 2:**
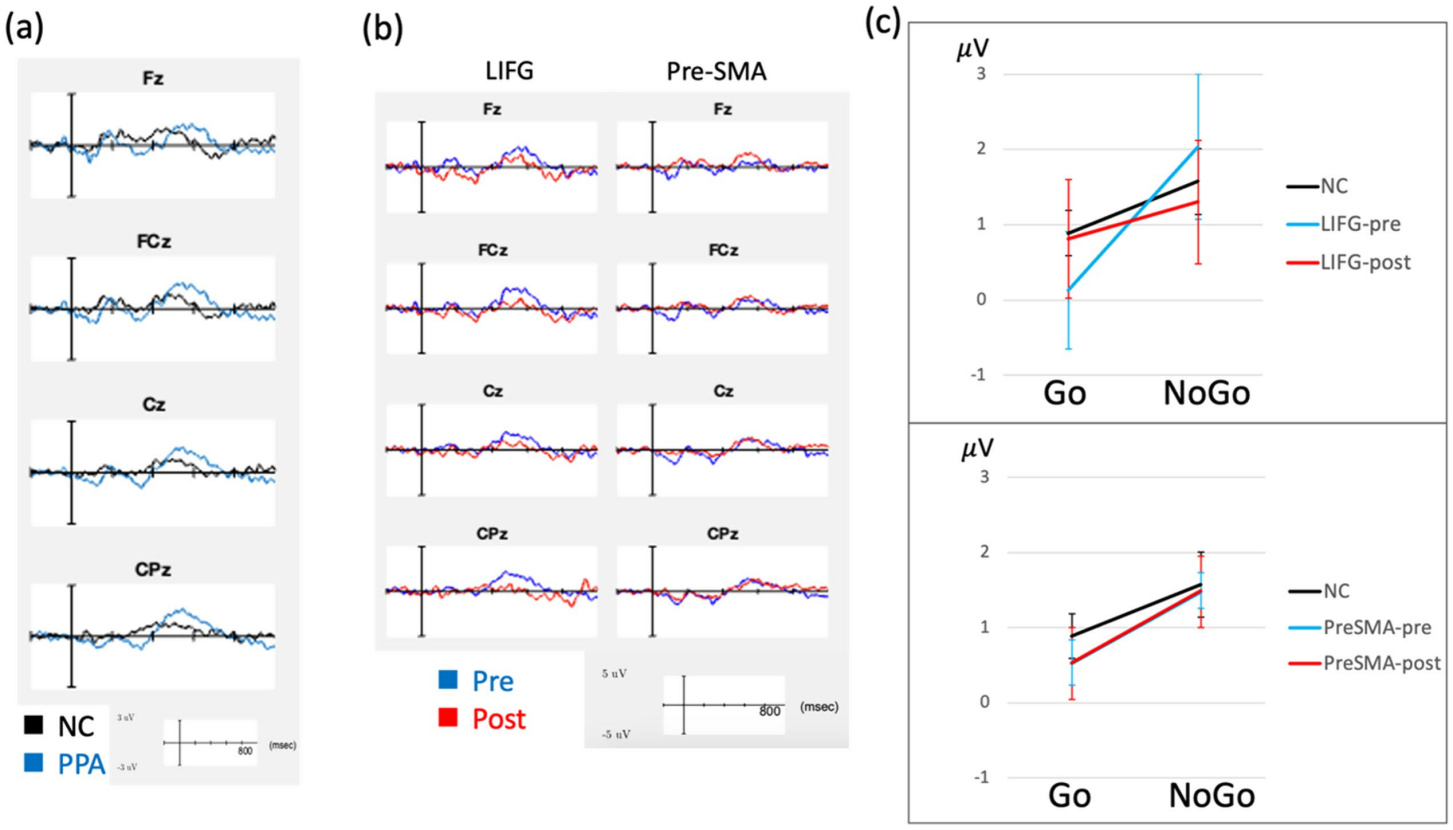
ERP results. ERP waveforms are demonstrated, showing PPA compared to normal controls (NC) at baseline **(a)** and treatment related changes from baseline separately for each stimulation group **(b)**. These waveforms represent difference waves (Nogo minus Go) are averaged across both SC and OA versions of the Go-Nogo task. The group averaged P3 mean amplitude for Go and NoGo trials (averaged across SC and OA) is shown in **(c)**, comparing LIFG and pre-SMA separately to NC, with error bars representing standard errors. These waveforms did not include PPA08 due to missing post-treatment data.

During these Go/NoGo tasks, subjects were instructed to push a button for certain stimuli (Go) while withholding responses for others (NoGo). The single car task (SC) involved basic categorization and used single exemplars of a car (Go) and a dog (NoGo). The object animal task (OA) involved superordinate categorization and used multiple exemplars of objects (Go) and animals (NoGo) across trials. Each task consisted of 200 trials: 160 (80%) ‘Go’ trials that required a response through button pressing and 40 (20%) ‘NoGo’ trials that required inhibition/withholding of a response (57). Concurrent EEG data were recorded from a 64-electrode Neuroscan Quikcap using a Neuroscan SynAmps2 amplifier and Scan 4.5 software (sampling rate: 1 kHz, DC-200 Hz). Trials were included for ERP analysis only if they were correct responses. Baseline correction was done by subtracting the mean amplitude of the pre-stimulus interval (−200 ms to 0 ms) from each time point. Individual ERPs were generated by averaging all the included epochs for Go and NoGo. Difference waves were also generated (NoGo minus Go epochs) for each individual. See Supplementary material for the detailed EEG preprocessing methods. We focused on N2/N200 and P3/P300 components around the midline electrodes. Electrode sites and time windows were selected *a priori* based on previous N200/P300 studies (38, 57, 58). For both Go and NoGo trials, we first measured peak latency between 150 and 300 ms for N200 at electrodes Fz-FCz-Cz, and between 250 and 650 ms for P3 at electrodes FCz-Cz-CPz. We used the peak latencies from all subjects’ baseline data for each group separately for each task (NC vs. PPA, SC vs. OA) and calculated mean amplitude within this window (mean latency ± 1 SD, separately for N200 and P300) to better estimate amplitude differences. We then averaged mean amplitude and peak latency across electrodes Fz, FCz, and Cz for examining N200 and across the electrodes FCz, Cz, and CPz electrodes for examining P300. This approach has been used and published in our previous studies (38, 58). Group level ERPs (NoGo – Go) are illustrated in Figure 2, and group level ERPs for Go and NoGo trials with topographies are illustrated in Supplementary Figures 1–3.

### Statistical analysis for primary outcome measures

To evaluate effect of intervention, raw scores from primary outcome measures (phonemic fluency—FAS, category fluency—animals, BNT, WPM, MC, and CoreLex) were evaluated using linear-mixed effect models (LMMs) for each dependent variable with one between-subject factor of target (Group/stimulation condition: pre-SMA versus LIFG) and one within-subject factor of time (baseline versus post-treatment/follow-up) with a random intercept for subject to account for repeated-measures. LMMs were used to maximize statistical power given the small sample size within each group (*n* = 4) to account for within-subject variability and better assess group x time interactions. LMMs were conducted separately to evaluate immediate effects of treatment and potential maintenance of treatment gains at 8-weeks as compared to baseline measures. Standardized t-scores and z-scores were also reported for each individual participant for baseline, immediate post, and 8-weeks post in Supplementary Table 1. Clinical significance was measured using standardized assessments, normed for individuals with similar, age, sex, and levels of education. Clinically meaningful change was set at a change of at least 1 in z-score or 10 in t-score in the follow-ups compared to baseline and reported for primary outcome measures (15), which is a conservative threshold equivalent to improving at least two clinical interpretations for performance (e.g., moderately impaired to mildly impaired). All analyses were conducted using R (version 4.3.2) (59). LMMs were fit using the lmer() function from the lme4 package, Type III Wald chi-square tests were conducted to assess the significance of main effects and interactions, using the Anova() function from the car package (60). For additional fixed effects estimation, *p*-values and degrees of freedom estimated using the lmerTest package (version 3.1.3) (61). *Post hoc* comparisons were performed using the emmeans package (version 1.10.0) (62), with Tukey corrections applied to adjust for multiple comparisons. Given the small sample size, effect sizes were interpreted alongside 95% confidence intervals, and Hedges’ *g* was calculated to provide a bias-corrected estimate of standardized mean differences (63) and was computed using the effectsize package (version 0.8.9) in R (64). An alpha level of 0.05 was used for determining statistical significance.

### Statistical analysis for EEG task performance and ERP measures

For baseline performance, LMMs were administered to examine group difference (control vs. PPA), task effect (SC vs. OA) and their interaction in (1) Go-RT, (2) Go-accuracy, (3) NoGo-accuracy, (4) N2 latency, (5) N2 amplitude, (6) P3 latency, and (7) P3 amplitude [all of the ERP difference wave (NoGo – Go)] using R, with an alpha level set at 0.05 for significance. For treatment effects, LMMs were administered to examine target assignment (pre-SMA vs. LIFG), treatment effects (baseline vs. immediate-post), task effect (SC vs. OA) and their interactions in (1) Go-RT, (2) Go-RT, (3) NoGo-accuracy, (4) N2 latency, (5) N2 amplitude, (6) P3 latency, and (7) P3 amplitude [all of the ERP difference wave (NoGo – Go)].

## Results

### Primary outcome measures

All group means and standard deviations for primary and secondary outcome measures for each time point are provided in Table 2. In all separate LMMs, no significant between group effects were observed for any of the primary outcome measures (*p* > 0.05) so the following results report on the within-subject main effects of time and interactions.

**Table 2:** Primary outcome measures means and standard deviations per group by time.

Measure	Pre-SMA			LIFG		
	Baseline	Immediate	8-week	Baseline	Immediate	8-week
COWAT	19.3 (11.6)	19.5 (11.5)	23.5 (9.7)	21.0 (11.6)	**29.0 (11.5)** ^†^	30.3 (9.8)
Animals	8.3 (16.5)	9.8 (4.9)	11.8 (4.5)	12.5 (4.9)	15.8 (4.9)	13.5 (4.5)
BNT	20.0 (7.5)	21.8 (5.4)	18.3 (9.3)	23.5 (7.5)	23.8 (5.4)	22.0 (9.3)
WPM	63.1 (30.7)	65.9 (24.2)	79.5 (29.8)	50.5 (30.7)	54.5 (24.2)	61.5 (29.8)
MC	10.8 (5.1)	14.0 (5.6)	17.0 (5.0)*	12.0 (5.1)	14.5 (5.6)	15.0 (5.0)*
CoreLex	14.5 (3.9)	16.5 (3.2)	17.3 (2.2)	14.3 (3.9)	16.8 (3.2)	14.0 (2.2)

*Significant effects are highlighted in bold. *Indicates a significant effect for time; ^†^Indicates a significant interaction for time × group.

For immediate effects, there was a significant time x condition interaction in phonemic fluency (FAS) outcome measure revealed from a Type III Wald chi-square test, (*χ*^2^(1) = 8.31, *p* = 0.004), indicating that changes over time differed by stimulation condition. *Post hoc* comparisons revealed that participants receiving LIFG stimulation produced, on average, 8 more words immediately following treatment (*SE* = 1.9, *p* = 0.006), corresponding to a large, statistically reliable effect (Hedges’ *g* = 2.98, 95% CI [0.60, 5.35]). No significant change was observed from those receiving pre-SMA stimulation (*mean difference* = −0.25, *SE* = 1.9, *p* = 0.900), indicating the improvement was specific to LIFG stimulation. There were no significant main effects of time (*χ*^2^(1) = 0.017, *p* = 0.895) or condition (*χ*^2^(1) = 0.046, *p* = 0.830). No other effects were significant for category fluency, BNT, WPM, CoreLex, or MC in terms of immediate post-treatment difference from baseline and time *x* condition interaction (all *ps* > 0.05).

For longer-term effects at 8-week follow-up, phonemic fluency no longer showed a significant time x condition interaction (*p* = 0.305), indicating both groups performed similarly. However, a significant improvement was observed in main concept (MC) scores (*χ*^2^(1) = 15.46, *p* < 0.001), indicating that participants improved from baseline to 8-weeks post treatment regardless of stimulation condition. *Post hoc* comparisons confirmed a significant increase in MC scores from baseline to 8 weeks (*mean difference* = 4.6, *SE* = 1.12, *p* = 0.006) with a corresponding large and statistically reliable effect-size (Hedges’ *g* = 2.06, 95% CI [0.40, 3.73]). No significant main effect of condition or interaction was observed (*ps* > 0.15). There was no significant main effect of condition on MC scores (*χ*^2^(1) = 0.12, *p* = 0.735) and no significant interaction (*χ*^2^(1) = 2.06, *p* = 0.151). No other significant main effects or interactions were observed for phonemic or category fluency, BNT, WPM, or CoreLex measures in terms of 8-week change from baseline (all *ps* > 0.05).

### Clinically meaningful change of primary outcome measures

Given that statistical significance cannot be equated to clinically meaningful change, we calculated the percentage of PPA patients who made clinically meaningful improvement at the 2 follow-up time points (Table 3; see individual data in Supplementary Table 2). None showed clinically meaningful change in BNT, consistent with statistical findings. In addition, only the LIFG group (25% at immediate follow-up and 50% at 8-week follow-up) showed clinically meaningful change in phonemic fluency (COWAT), again consistent with statistical findings. In contrast, despite no statistical significance in category fluency (Animals), 2 patients in the LIFG group showed clinically meaningful change at immediate post follow-up and 2 each in the pre-SMA and LIFG groups showed clinically meaningful change at 8-weeks follow-up. We were unable to calculate this for WPM and discourse measures because standardized scores cannot be derived.

**Table 3:** Clinically meaningful change after treatment.

Measures	Pre-SMA (*n* = 4)		LIFG (*n* = 4)		All (*n* = 8)	
	Immediate	8 weeks	Immediate	8 weeks	Immediate	8 weeks
BNT	0	0	0	0	0	0
COWAT	0	0	1 (25%)	2 (50%)	1 (13%)	2 (25%)
Animals	0	2 (50%)	2 (50%)	2 (50%)	2 (25%)	4 (50%)

Improved by at least T of 10 or Z of 1.

### EEG task measures for NC vs. PPA at baseline

**Figure 3:**
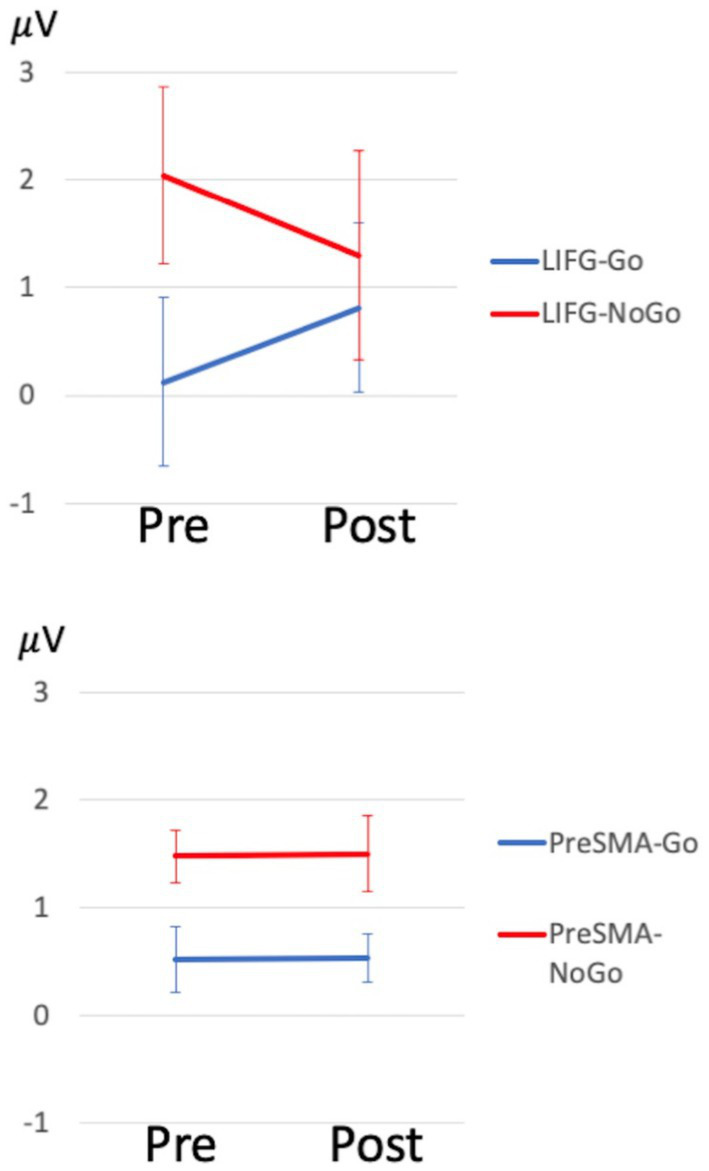
ERP changes between stimulation groups. ERP waveforms are demonstrated. The group averaged P3 mean amplitude for Go and NoGo trials (averaged across SC and OA) is shown to compare baseline (Pre) and post-treatment (Post) data in LIFG and pre-SMA, separately. Error bars represent standard errors. These waveforms did not include PPA08 due to missing post-treatment data.

For RT, LMMs showed significant main effects of task (*χ*^2^(1) = 6.41, *p* = 0.011, Hedges’ *g* = 1.42, 95% CI [0.51, 2.32]) and group (*χ*^2^(1) = 4.94, *p* = 0.026, Hedges’ *g* = 2.27, 95% CI [0.03, 4.51]), while interaction between task and group was not significant (*χ*^2^(1) = 0.03, *p* = 0.866). Go-RT was faster in control than PPA (425 ± 140.8 < 594 ± 140.9 ms) and faster in SC than OA (457 ± 151 < 562 ± 151 ms). For other measures (accuracy and ERP measures), LMMs showed significant task effects in P3 latency (*χ*^2^(1) = 8.94, *p* = 0.003, Hedges’ *g* = 0.96, 95% CI [0.12, 1.81]); SC, 493 ms, shorter than OA, 537 ms. No other main effects were significant for any other measures (*ps* > 0.05). P3 latency data also showed a reliable trend in task x group interaction (*χ*^2^(1) = 3.01, *p* = 0.083) and *post hoc* comparisons revealed P3 latency significantly differed between tasks in the NC group, indicating OA > SC P3 latency in the NC group (*mean difference* = 73.1 ms, *SE* = 24.5, *p* = 0.011) which showed a large and statistically reliable effect size (Hedges’ *g* = 1.60, 95% CI [0.12, 1.81]), with no task differences in P3 latencies observed in the PPA group (*p* = 0.525). There was also a significant task x group interaction in N2 latency (*χ*^2^(1) = 4.28, *p* = 0.039). This was explained by SC > OA N2 latency in NC (293 vs. 256 ms) but SC < OA N2 latency in PPA (264 vs. 288 ms), although these differences were not significant in *post-hoc* comparisons (*ps* > 0.05). All other 2-way and 3-way interactions were not significant for any other measures (all *ps* > 0.05). Group level EEG task performance and ERP measures are reported in Supplementary Table 3. ERP waveforms are demonstrated in Figures 2, 3.

### EEG task measures for PPA pre and post HD-tDCS

For task performance, LMMs revealed only main effects of task in Go-RT (*p* = 0.031, Hedges’ *g* = 1.35, 95% CI [0.45, 2.26]) showing faster RT in SC compared to OA (541 ms versus 628 ms, respectively). No other main effects or interactions, including treatment effects, were significant for other task performance measures (all *ps* > 0.05). For ERP measures, LMMs revealed main effects of treatment in P3 amplitude of the difference wave (*χ*^2^(1) = 8.84, *p* = 0.003); showing P3 amplitude was reduced from baseline at 1.17 mV to post-treatment at 0.57 mV. No other main effects were significant in any other measures (all *ps* > 0.05). There was also a reliable trend in treatment x target assignment interaction in P3 amplitude (*χ*^2^(1) = 3.38, *p* = 0.066). *Post hoc* comparisons showed a significant decrease of P3 amplitude in the LIFG group from baseline to post-treatment (from 1.39 mV to 0.18 mV, *p* = 0.018, Hedges’ *g* = 1.49, 95% CI [0.19, 2.80]) but no significant change in the pre-SMA group (from 0.96 mV to 0.96 mV, *p* = 0.991). No other two-way or three-way interactions were significant for any other measures. Group level task performance and ERP measures are reported in Supplementary Table 3 and ERP waveforms are illustrated in Figure 2. To further evaluate this treatment effect in P3 amplitude, we compared qualitatively control data to PPA data separately for the LIFG and pre-SMA groups (Figure 2). The LIFG group demonstrated larger difference between Go and NoGo P3 amplitude at baseline, while this difference was reduced and qualitatively more similar to normal at post-treatment testing (Figures 2, 3). In contrast, the pre-SMA group did not demonstrate evidence of a change between Go and NoGo P3 amplitude from baseline to post-treatment follow-up (Figures 2, 3).

### Safety and tolerability of HD-tDCS in PPA

This pilot study aimed to assess not only preliminary treatment effects but also the feasibility, tolerability, and safety of administering HD-tDCS at two target locations (pre-SMA, LIFG) in individuals with PPA. Safety and tolerability of anodal HD-tDCS stimulation at 1 mA was monitored across treatment sessions. All participants received active stimulation and across 80 treatment sessions (8 participants × 10 sessions), adverse effects were generally mild and transient. No serious adverse events such as seizures or psychotic symptoms were reported during our study. Adverse events were documented via questionnaire, with the most frequently reported effects being itching (16 out of 80 treatments; 20%) and tingling (15 out of 80 treatments; 19%). Sleepiness was reported in 5 sessions (6%), and headache occurred in 3 sessions (4%). Dizziness and migraine each occurred in 3 sessions (4%), with 2 out of 3 migraines localized to the stimulation site. Nausea was reported in 2 sessions (3%). Isolated instances of acute mood changes, burning sensations, skin redness, and mid-nocturnal insomnia were each reported in one treatment session (1%) across different participants. All adverse effects were self-limited, resolved without medical intervention, and no participants discontinued treatment due to discomfort. Although one participant showed reduced tolerance to complete the study, this was attributed to difficulty tolerating changes in routine rather than any reported discomfort related to stimulation. Because the participant withdrew from the study, any potential subjective cognitive decline related to stimulation could not be established. These findings align with a systematic review of 209 tDCS studies (65), which reported no significant differences in adverse event rates between active and sham stimulation. The most commonly reported effects (i.e., itching, tingling, headache, discomfort, and burning sensation) occurred at comparable rates with those observed in the present study.

## Discussion

In this pilot study, we found that HD-tDCS targeting different brain regions elicited differential effects in word retrieval measures as well as in EEG responses. We also showed that the protocol was well-tolerated in PPA patients at earlier stages with no severe adverse effects.

HD-tDCS improved verbal fluency in both stimulation groups. However, effects were differentially improved in LIFG relative to pre-SMA stimulation. Phonemic verbal fluency (i.e., FAS) significantly improved immediately following LIFG stimulation with no immediate change in performance for those who received pre-SMA stimulation. However, statistically significant gains were not maintained for the LIFG group at 8-week follow-up. This is in contrast with regards to clinical significance, which showed 25% and 50% of the subjects in the LIFG group achieved clinically meaningful improvement in phonemic fluency immediately and 8-weeks post-treatment, respectively. For category fluency, despite no statistically significant findings, it is notable that 50% of the LIFG group showed clinically meaningful improvement immediately and 8-weeks post-treatment, while 50% of the pre-SMA group showed clinically meaningful improvement in 8-weeks post-treatment. To summarize, LIFG stimulation induced immediate improvement in both phonemic and category fluency that sustained after 8 weeks, while pre-SMA induced delayed improvement after 8 weeks in these same measures. Our prior work using HD-tDCS targeting the pre-SMA in individuals with chronic traumatic brain injury also showed this delayed effect in category fluency (28, 29). More adaptive, off-line, responses to tDCS have been elaborated (20) and we posit that such processes involving the underlying neural circuits for category fluency could result in such delayed effects, although future study is warranted to address this mechanism especially in the context of a neurodegenerative disease.

There was also a statistically reliable significant effect in the MC discourse measure in both stimulation groups at the final 8-week follow-up. Discourse production improved in completeness and accuracy by 7 points from pre-SMA stimulation and by 3 points from LIFG stimulation (see Table 2). This delayed effect, driven by the pre-SMA group, aligns with the clinically significant gains in category fluency observed at the 8-week follow-up, further reinforcing the pattern of late-emerging benefits associated with pre-SMA stimulation consistent with previous findings (20, 28, 29). Other primary language outcome measures (WPM, CoreLex, and BNT) showed no effect of treatment at either time-point for either treatment group, possibly reflecting reduced sensitivity or limited statistical power to detect changes.

LIFG stimulation also induced improvements in non-language tasks including working memory and processing speed (Supplemental material), while pre-SMA stimulation did not induce statistically significant change in any non-language tasks, in contrast to our original hypothesis. It is possible that stimulating the LIFG could modulate both the underlying domain general and domain specific neural circuits. Another possibility is that HD-tDCS, despite its better focality, could still affect more brain regions than had been targeted, causing more diffuse effects in the prefrontal regions. It is therefore of future interest to examine other non-linguistic functions as outcome measures in the study design for PPA patients and explore their impact on daily functioning, which is beyond the scope of the current study.

These above behavioral findings are accompanied by changes in frontal P3 amplitude most evidently in those who received LIFG stimulation, showing a likely normalization of brain activity, but not observed in those who received pre-SMA stimulation. This was an unexpected finding as we initially hypothesized to observe more frontal changes elicited by pre-SMA stimulation, given that pre-SMA is thought to be part of the neural generators for N2/P3 ERP responses during the Go-NoGo tasks. One explanation could be that the LIFG group showed an ERP pattern at baseline more dissimilar to controls (see Figure 2), which could be indicative of more advanced pathological change (much larger NoGo than Go P3 amplitude, compared to control), implying greater capacity to be modulated by tDCS. In contrast, the pre-SMA group had an ERP pattern at baseline more similar to controls (see Figure 2). This might imply that baseline ERP patterns are predictive of treatment response to stimulation, as we did see greater effects in the LIFG group, in both word retrieval and other cognitive measures. We also posit that by stimulating the LIFG, the pre-SMA may also be affected downstream as these two regions have an intricate connection both functionally and structurally (27, 66), leading to changes in midline frontal ERP. An alternative hypothesis is that modulating the LIFG may potentially affect the right IFG, via the reciprocal activity between the homologs across the hemispheres (67, 68) leading to changes in the neural circuit involving the pre-SMA during inhibition and selection functions (69, 70). The current study is not powered to discern whether the group effect is due to differences in stimulation targets or in patient baseline characteristics. Future studies examining a larger sample and/or including subjects who will undergo both pre-SMA and LIFG protocols will be required to directly investigate this question. Despite this limitation, few studies have examined neural changes in response to tDCS in PPA (12) and our study is the first to use EEG measures to monitor tDCS effects in PPA patients.

We acknowledge several limitations of the current study. We did not include sham control, as this was an open label study with a small sample. It is possible that some of the observed differences could represent practice effects. Future research may mitigate potential practice or placebo effects by incorporating a double-blind randomized sham controlled cross-over design to compare effects of pre-SMA vs. LIFG, where each subject will be randomly assigned to one of the three potential conditions (active pre-SMA, active-LIFG, or sham) in a randomized order. A study design as such will allow us to more directly compare effects within subjects, despite the limitation that the subsequent could be influenced by the prior conditions if the wash-out period is not sufficient. We recognize that other factors such as psychiatric comorbidities, medication use, and sleep apnea could be affecting the results. Although we do not think they play a substantial role in confounding the results, future larger trials can consider including these factors in their analyses to account for their potential influences. We also recognized that we did not include svPPA, with one such patient being excluded from study due to severe impairment, so the findings are not applicable to those with a diagnosis of svPPA. This study also focused on early stage PPA and patients with higher severity of symptoms were excluded from the study and thus we are unable to determine the tolerability or estimate effects of stimulation in more advanced stages of PPA.

Lastly, we did not collect brain MRI data for this pilot trial. Our future study design will consider incorporating structural brain MRI analysis, such as volumetric assessments of brain regions commonly affected in PPA, to provide additional information that can inform behavioral outcomes from brain stimulation. Furthermore, we did not pursue analysis on electrophysiological data using time-frequency methods to investigate changes in different oscillatory activities (theta, alpha, gamma) because this was not part of the initial goal for this pilot study. Future studies should also consider these measures as oscillatory changes could be pertinent to the underlying pathology such as Alzheimer’s disease and correlate with behavioral changes in response to intervention. This may further provide circuit-level changes relevant to cognitive function in PPA, as shown in prior electrophysiological studies (71).

In conclusion, the present study provides preliminary evidence to support HD-tDCS targeting the LIFG, without concurrent speech therapy, to improve word retrieval deficits in individuals with early stage PPA. Findings also support the pre-SMA as a potential alternate stimulation site, offering additional evidence of improved word retrieval and associated neural changes in semantic selection and inhibition processes, as measured by the Go/NoGo EEG task. These findings may also provide implications for individuals unable to tolerate conventional speech/language intervention (i.e., fatigue, frustration, profound deficits, etc.) and future research explore this approach to expand treatment options for addressing word retrieval deficits in these populations. Although additional research is warranted to confirm these effects, measures of clinical significance may provide additional support for the stimulation effects on the individual-level compared to conventional analysis of variance methods. The optimal duration, frequency, dosage, and other parameters of stimulation remain to be settled, and may ultimately depend on what outcome measures are targeted for which patient population (disease severity, PPA subtypes, clinical characterization, etc.).

### Data availability statement

The datasets presented in this study can be found in online repositories. The names of the repository/repositories and accession number(s) can be found in https://osf.io/b73cn/.
