# Supplementary material for: High-definition brain stimulation targeting separate regions leads to differential word retrieval outcomes in patients with primary progressive aphasia: a pilot study
Source: Front Neurol. 2025 Sep 17;16:1630103. doi: 10.3389/fneur.2025.1630103 (PMC12484069; doi:10.3389/fneur.2025.1630103)
Supplement: Supplementary file 1 [file Supplementary_file_1.docx]

**SUPPLEMENTARY MATERIALS**

**Supplementary Methods: Participant medical history**

Participants enrolled in the study each completed medical history form to document an overview of co-morbid medical conditions, family history, and prescribed medications at the time of the study. Summary of each participants medical history is provided in supplementary table 1.

**Supplementary Methods: Discourse methods**

Methods of discourse analysis, including main concept (MC) and core lexicon (CoreLex) analyses, have been established to evaluate connected speech samples elicited from the cookie theft description task (Dalton et al., 2024), indicating a total possible MC score of 30 and a total of 26 CoreLex targets used to evaluate discourse samples in this study. MC measures informativeness -- both micro-linguistic features of the discourse sample (i.e., below sentence level) such as information units (i.e., lexical targets) and macro-linguistic features (i.e., sentence level and above) such as relevant verb or noun phrases and subordinate clauses (Richardson & Dalton, 2016; 2020; Kim et al., 2021). The reliability of main concept analysis has been previously evaluated (Kong, 2009), demonstrating acceptable inter-rater, intra-rater, and test–retest reliability in individuals with aphasia, averaging 0.80, and strong concurrent validity with other language measures, including spontaneous speech (r = 0.94) and fluency (r = 0.92). CoreLex evaluates micro-linguistic features in discourse where a previously normed checklist for targets is provided for a given discourse prompt (Dalton et al., 2024). Words per minute were estimated from connected speech samples elicited from the picture description task to evaluate potential changes in speech rate following stimulation. Filler words (i.e., um), disfluencies (i.e., part word/ whole word repetitions) were omitted from the total count. Words were calculated per minute and averaged to provide total estimated WPM.

**Supplementary Methods: EEG recording and preprocessing**

Concurrent EEG data were recorded from a 64-electrode Neuroscan Quikcap using a Neuroscan SynAmps2 amplifier and Scan 4.5 software (sampling rate: 1 kHz, DC-200 Hz). EEG channels with impedance exceeding 10 kW were discarded from further processing. Poorly functioning electrodes were also excluded manually by visual inspection of the raw data as well as spectrogram. On average, data from fewer than 3% of electrodes were rejected and data for these electrodes were interpolated (see Chiang et al., 2024 for detail). The continuous EEG data were high-pass filtered at 0.5 Hz followed by low-pass filtered at 40 Hz using a finite impulse response filter. The filtered EEG data then underwent independent component analysis (ICA) for artifact removal using EEGLab (Delorme and Makeig, 2004; ICLabel; Pion-Tonachini et al., 2019). ICA components representing artifacts not identified previously by the algorithm were removed manually to complete data cleaning. EEG data were segmented per trial into multiple EEG epochs (-200 to 1000ms, time-locked to the stimulus onset). Epochs having amplitude of more than 75 mV were rejected and epochs with extreme values were excluded by rejection algorithms in EEGLAB. The EEG data were re-referenced to the average potential over the entire scalp. Trials were included for ERP analysis only if they were correct responses and for Go with RT between 100 and 1500ms. Baseline correction was done by subtracting the mean amplitude of the pre-stimulus interval (–200 ms to 0 ms) from each time point. Individual ERPs were generated by averaging all the included epochs for Go and NoGo. Difference waves were also generated (NoGo minus Go epochs) for each individual. On average, more than 128 Go trials and 31 NoGo trials were entered for each individual among control and PPA individuals.

**Supplementary Methods for secondary outcome measures**

Assessments of other cognitive domains included Trails-Making Tests A & B (TMT-A & B, Partington & Leiter, 1949), the Digit Symbol Substitution Test (DSST; Weschler, 2008), Digit Span Forward & Backward (DSF/DSB; Weschler, 2008), the Rey-Osterrieth Complex Figure Test (Rey-O; Rey & Osterrieth, 1941), and the Hopkins Verbal Learning Test-Revised (HVLT-R, Benedict et al., 1991). Other psychosocial questionnaires included Geriatric Depression Scale (GDS, Sheikh & Yesavage, 1986) and Neuropsychiatric Inventory (NPI-Q, Kaufer et al., 2000) and scores provided for each participant in individual tables. Multiple versions were administered across time when available.

To evaluate effect of intervention, raw scores from secondary outcome measures were evaluated using linear mixed-effect models (LMM) with one between-subject factor of target (pre-SMA, LIFG) and one within-subject factor of time (baseline versus post treatment follow up). LMMs were conducted separately to evaluate immediate effects of treatment and potential maintenance of treatment gains at 8 weeks as compared to baseline measures. Psycho-social measures were not collected at all time points for every participant and were excluded from the analysis but provided in participant tables in supplementary material (Supplementary Table 3). All analyses were performed using R (version 4.3.2), with an alpha level set at 0.05 for significance. LMMs were fit using the lme4 and lmerTest packages, and post hoc comparisons were conducted using the emmeans package with Bonferroni corrections applied for multiple comparisons. Hedges’ *g* was used to estimate effect sizes, providing a bias-corrected measure of standardized mean differences appropriate for small sample sizes (Cumming, 2013).

**Supplementary Results for Secondary outcome measures**

All remaining group means and standard deviations for secondary outcome measures for each time point are provided in supplementary table 2. All between group effects for each measure were nonsignificant and not reported below (p>.05). Results are provided for significant secondary outcome measures within-subject main effects and interactions.

For immediate effects, a linear mixed-effects model revealed a significant Time × Condition interaction on DSST performance (*χ²*(1) = 5.96, *p* = .015), suggesting that changes in DSST scores over time differed by group (stimulation condition). Post hoc comparisons showed that participants in the LIFG group demonstrated a significant improvement in DSST performance from baseline to post-treatment (*mean difference* = 10.5, *SE* = 2.1, *p* = .002), corresponding to a statistically reliable large effect size (Hedges’ *g* = 3.54, 95% CI (0.94, 6.13)). In contrast, participants in the pre-SMA group did not show a significant change over time (*p* = .173). There was also a significant difference from baseline in DS-B with a significant time x group interaction (*χ²*(1) = 13.71, *p* < .001). Post hoc comparisons showed that participants in the LIFG stimulation group demonstrated significant improvement in DS-B scores from baseline (*mean difference* = 1.75, SE = 0.38, *p* = .004), corresponding to a statistically reliable large effect size (*Hedges’ g* = 3.24, 95% CI (0.76, 5.72)). In contrast, no significant change was observed in the pre-SMA group (*p* = .537). Additionally, a significant improvement in delayed visuospatial memory was observed on the Rey-O Delayed Recall (ReyODR), with a significant main effect of time (*χ²*(1) = 5.22, *p* = .022), suggesting potential gains from baseline across both stimulation groups. Although the corresponding effect size was moderate, (Hedges’ *g* = 0.46, 95% CI (-0.49, 1.40)), the confidence interval included zero, indicating the effect was not statistically reliable and should be interpreted with caution. Main effects and interactions for the remaining cognitive measures were non-significant at immediate post *(ps* >.05)*.*

For longer-term effects at 8-week follow-up, the LMMs revealed a significant time by group interaction on DS-B performance (*χ²*(1) = 25.00, *p* < .001). Post hoc comparisons indicated that the LIFG group maintained gains at 8-weeks post treatment from baseline (*mean difference* = 1.25, SE = 0.18, *p* < .001), corresponding to a statistically reliable large effect size (*Hedges’ g* = 5, 95% CI (1.76, 8.24)). Again, there was a sustained significant main effect of time in Rey-O Delayed Recall from baseline (*χ²*(1) = 4.06, *p* = .044), suggesting improvement across groups. Although the corresponding effect size was large, it was not statistically reliable (Hedges’ *g* = 1.70, 95% CI (–0.27, 3.66)). Additionally, there was a significant main effect of time on verbal list learning (HVLT-total) performance (*χ²*(1) = 5.18, *p* = .023), suggesting improvement across groups (*mean difference* = 3.38, SE = 1.32, *p* = .043). However, the corresponding large effect size was not statistically reliable (Hedges’ *g* = 1.28, 95% CI (–0.13, 2.69)). Lastly, a significant main effect of time on verbal list discrimination performance, as measured by the HVLT Discrimination score, was observed (*χ²*(1) = 6.05, *p* = .014), suggesting improvement across groups from baseline. However, the corresponding effect size for the time effect was large but not statistically reliable (Hedges’ *g* = 1.12, 95% CI (–1.23, 3.46)). DSST in terms of 8-week post-treatment difference from baseline interaction effects were not maintained (*p*=.140) and remaining cognitive measures continued to show no significant effects or trends (*ps >.*10).

**Supplementary table 1. Participant medical history**

| Subject | Age | Sex | Diagnosed Conditions | Surgery History | Smoking Status | Drinking Status | Family History of Dementia | Medications |
| --- | --- | --- | --- | --- | --- | --- | --- | --- |
| PPA01 | 79 | M | Arthritis, gout, osteoporosis, prostate disease | Back surgery, cosmetic surgery, eye surgery, knee surgery | No | Yes | Yes | Alfuzosin, Allopurinol, Donepezil, Dutasteride, Loratadine, Memantine, Timolol |
| PPA02 | 74 | M | High blood pressure | Meniscus repair, Appendectomy, Finger surgery | No | Yes | Yes | None |
| PPA03 | 69 | F | Depression | None | No | Yes | Yes | Exelon, Lexapro |
| PPA05 | 72 | M | High blood pressure, diabetes, high cholesterol, dementia, sleep apnea, dizziness, falling | None | No | No | *Not listed* | None |
| PPA07 | 74 | M | Heart disease (atrial fibrillation), hernias | Ablation for atrial fibrillation | No | Yes | No | Tykosiw, Eliquis |
| PPA08 | 68 | F | High blood pressure, high cholesterol, thyroid disease, cancer, headaches, arthritis | Knee replacement | No | Yes | No | Trospium chloride, Losartan, Trazodone, Zoloft |
| PPA09 | 75 | M | High cholesterol, depression/anxiety, prostate cancer, arthritis | Prostate surgery, Shoulder replacement | No | Yes | No | Trazodone, Losartan, Rosuvastatin, Carbidopa, Bupropion |
| PPA11 | 76 | F | Thyroid disease, dementia, cancer, headaches, Alzheimers, hypotension, dry mouth, allergies | Thyroidectomy | No | No | Yes | Levothyroxine, Memantine, Midodrine, Escitalopram, Loratadine |

**Supplementary table 2. Secondary outcome measures overall means (standard deviations) per group by time**

|  | ***Pre-SMA*** | | |  | ***LIFG*** | | |
| --- | --- | --- | --- | --- | --- | --- | --- |
|  | *Baseline* | *Immediate* | *8-week* |  | *Baseline* | *Immediate* | *8-week* |
| DSST | 21.0(13.1) | 24.3(12.2) | 23.3(12.9) |  | 32.3(13.1) | **42.8(12.2) ^†^** | 38.8(12.9) |
| DS-F | 4.8(1.3) | 4.5(1.2) | 5.0(1.2) |  | 5.0(1.3) | 4.5(1.2) | 5.8(1.2) |
| DS-B | 3.3(0.8) | 3.0(0.7) | 3.3(0.9) |  | 2.8(0.8) | **4.5(0.7) ^†^** | **4.0(0.9) ^†^** |
| ReyOCopy | 26.4(11.5) | 25.4(12.9) | 24.4(10.8) |  | 26.9(10.7) | 28.5(11.9) | 28.0(10.8) |
| ReyO ImR | 11.6(9.4) | 10.9(10.3) | 11.4(9.8) |  | 13.8(8.8) | 18.3(10.8) | 16(8.0) |
| ReyO DR | 9.9(8.5) | 14.1(10.2) | 13.1(9.0) |  | 12.1(8.5) | 16.8(10.8) | 16.0(9.0) |
| Trails A | 68.8(41.5) | 62.0(28.4) | 51.8(55.1) |  | 64.5(41.5) | 5.50.5(24.4) | 75.5(55.1) |
| Trails B | 248.8(104.0) | 195.5(113.7) | 190.3(112.3) |  | 149.8(104.0) | 143.5(113.7) | 137.3(112.3) |
| HVLT-Total | 11.3(7.4) | 12.8(9.1) | 15.5(8.5) |  | 19.0(7.4) | 21.3(9.1) | 21.5(8.5) |
| HVLT-Rec | 10.0(3.5) | 9.75(3.5) | 10.3(3.2) |  | 10.3(3.5) | 10.8(3.5) | 11.5(2.8) |
| HVLT-Disc | 6.25(3.6) | 6.50(5.3) | 9.62(4.1) |  | 9.25(3.6) | 10.25(5.3) | 10.50(3.6) |
| HVLT-DR | 3.8(2.9) | 4.5(3.7) | 4.5(4.3) |  | 7.3(2.9) | 7.8(3.7) | 7.5(4.3) |

Reliable effects are highlighted in bold. **^†^**significant interaction for time by group

**Supplementary Figure 1: ERP waveforms for normal controls (NC) vs PPA at baseline**


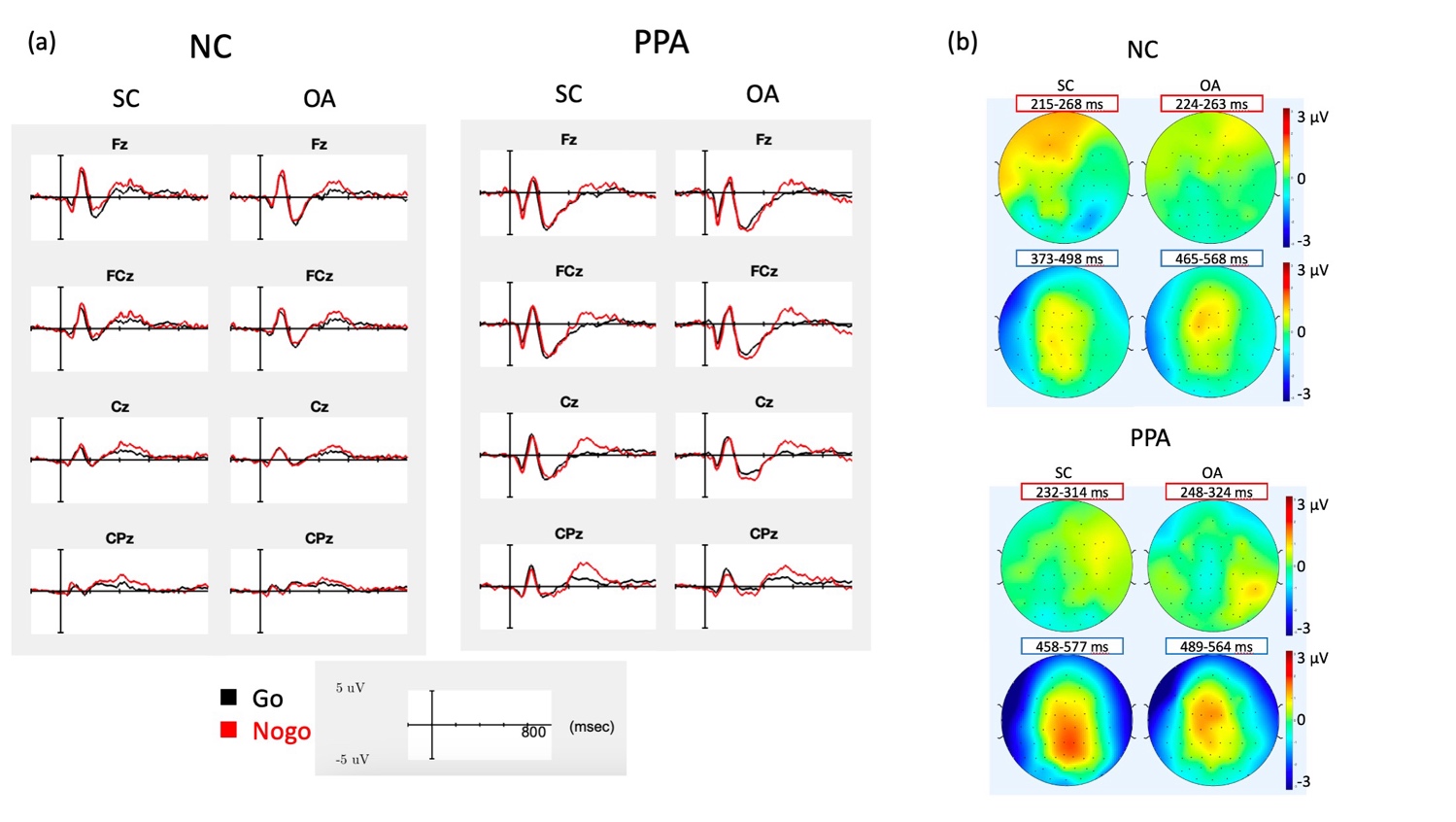


Group averaged ERP waveforms are depicted for normal controls (NC) and PPA (at baseline) separately for each condition (Go versus Nogo) and task (SC versus OA) (a). Averaged ERPs across each N2 (red boxes) and P3 (blue boxes) windows are represented topographically for NC and PPA (at baseline) (b).

**Supplementary Figure 2: ERP separately for Go and NoGo in LIFG group pre and post HDtDCS treatments**


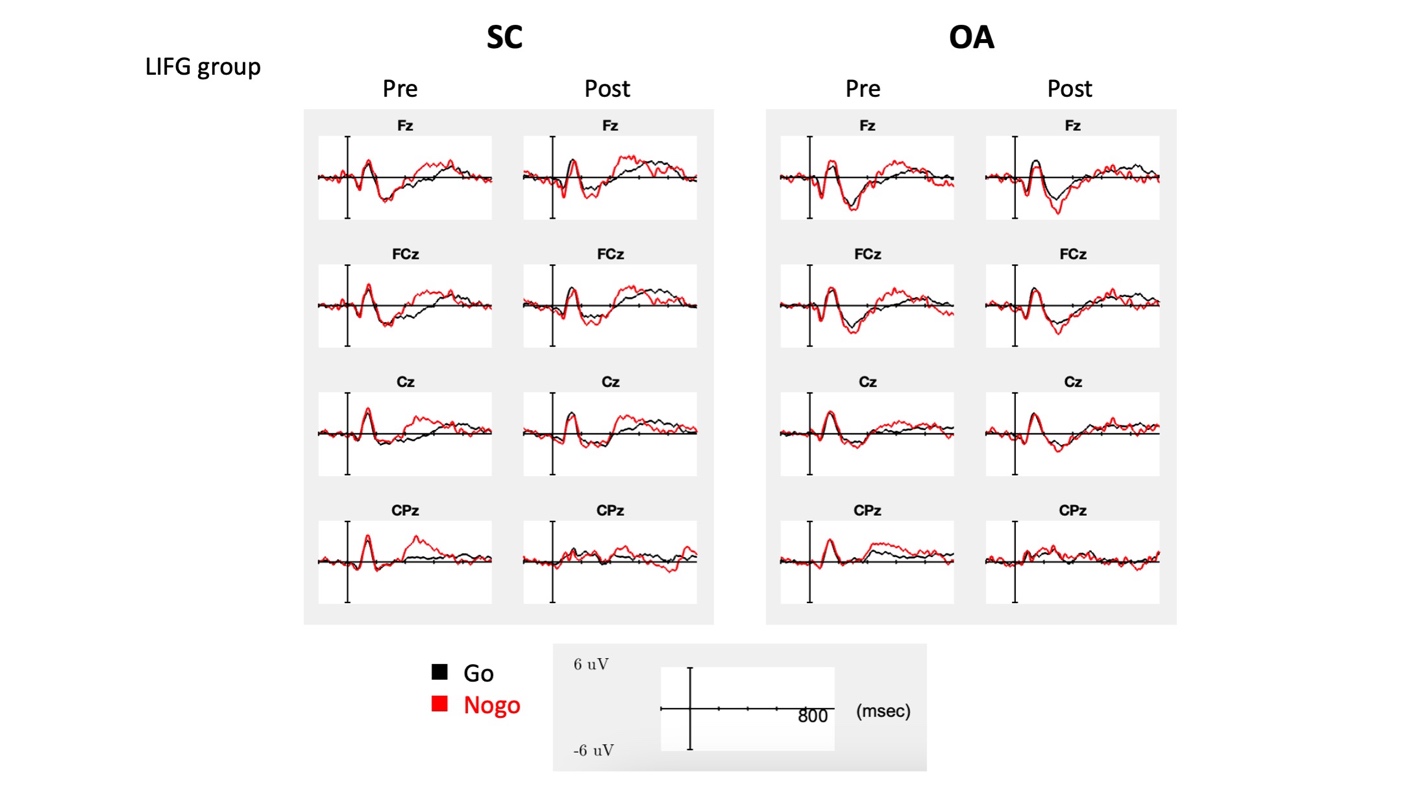


**Supplementary Figure 3: ERP separately for Go and NoGo in pre-SMA group pre and post HDtDCS treatments**


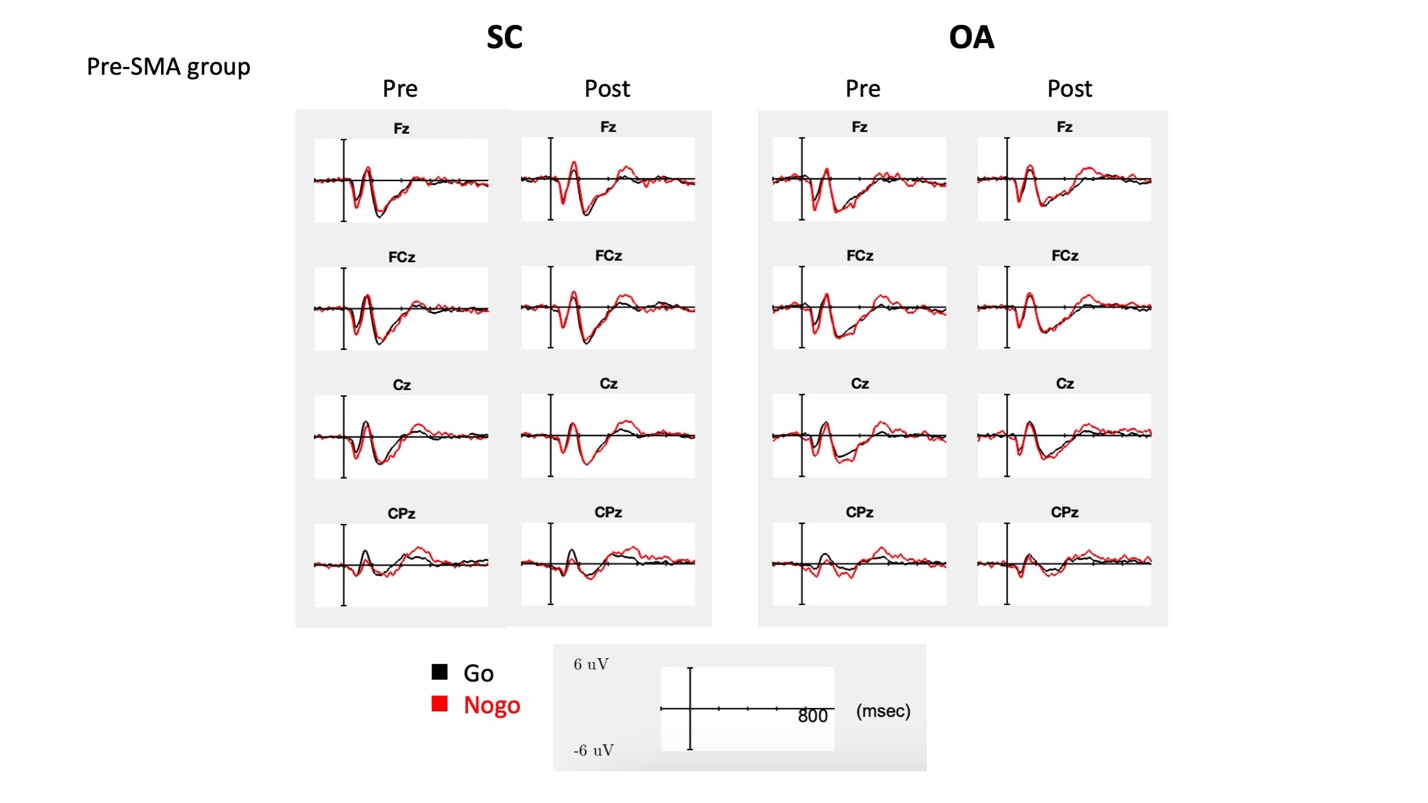


**Supplementary Tables 3: Participant pre-post standardized score comparison (individual)**

| **PPA01 (pre-SMA)** | **Baseline** | **Post** | **Follow up** |
| --- | --- | --- | --- |
| BNT-2 (T) | 59 | 51 | 67 |
| PPT (Total/52) | 48 | 51 | 50 |
| COWAT “FAS” (T) | 44 | 37 | 44 |
| Category fluency “Animals” (T) | 46 | 51 | 46 |
| **Executive Function** |  |  |  |
| Trails A/ B (T) | 45/51 | 45/51 | 41/46 |
| Digit span backward (Z) | -1.4 | -1.4 | -1.4 |
| Digit symbol substitution (Z) | -0.4 | -0.5 | -0.55 |
| **Working Memory** |  |  |  |
| Digit span forward (Z) | -1.5 | -1.5 | -1.5 |
| **Visuospatial construction** |  |  |  |
| Rey-O copy (T) | 48 | 58* | 48 |
| **Visual memory** |  |  |  |
| Rey-O immediate recall (T) | 79 | >80 | >80 |
| Rey-O delayed recall 15 mins (T) | 70 | >80* | >80 † |
| **Verbal memory** |  |  |  |
| HVLT-Trials 1-3 (T) | 39 | 56** | 57 † |
| HVLT-Delay 20 mins (T) | 42 | 57* | 56 † |
| HVLT-Retention (T) | 57 | 52** | 52 † |
| HVLT-Recognition (Total/12) | 9 | 12 | 12 |
| HVLT-Discrimination (T) | ≤20 | 62 | 56 |
| **Psychological Functioning** |  |  |  |
| GDS (total score) | 2 | 2 | 2 |
| NPIQ (total score) | 1 | 2 | 3 |

| **PPA02 (pre-SMA)** | **Baseline** | **Post** | **Follow up** |
| --- | --- | --- | --- |
| BNT-2 (T) | 29 | 33 | 29 |
| PPT (Total/52) | 50 | 48 | 48 |
| COWAT “FAS” (T) | 25 | 29 | 33 |
| Category fluency “Animals” (T) | 24 | 24 | 28 |
| **Executive Function** |  |  |  |
| Trails A/ B (T) | 53/NA | 53/52* | 53/52* |
| Digit span backward (Z) | -1.5 | -1.5 | -1.5 |
| Digit symbol substitution (Z) | -1.3 | -0.7 | -1.0 |
| **Working Memory** |  |  |  |
| Digit span forward (Z) | -3.4 | -3.4 | -2.5 |
| **Visuospatial construction** |  |  |  |
| Rey-O copy (T) | 55 | 55 | 50 |
| **Visual memory** |  |  |  |
| Rey-O immediate recall (T) | 49 | 55 | 61* |
| Rey-O delayed recall 15 mins (T) | 49 | 65* | 57 |
| **Verbal memory** |  |  |  |
| HVLT-Trials 1-3 (T) | <20 | <20 | 27* |
| HVLT-Delay 20 mins (T) | 27 | 27 | 27 |
| HVLT-Retention (T) | 41 | ≥80* | 33 |
| HVLT-Recognition (Total/12) | 9 | 5 | 8 |
| HVLT-Discrimination (T) | 21 | ≤20^ | 29 |
| **Psychological Functioning** |  |  |  |
| GDS (total score) | 3 | 3 | 6 |
| NPI-Q (total score) | 7 | 2 | 3 |

| **PPA03 (pre-SMA)** | **Baseline** | **Post** | **Follow up** |
| --- | --- | --- | --- |
| BNT-2 (T) | 22 | 30 | 18 |
| PPT (Total/52) | 46 | 45 | 46 |
| COWAT “FAS” (T) | 35 | 35 | 39 |
| Category fluency “Animals” (T) | 10 | 14 | 22* |
| **Executive Function** |  |  |  |
| Trails A/ B (T) | 17/NA | 17/NA | 31*/NA |
| Digit span backward (Z) | -1.24 | -1.2 | -1.2 |
| Digit symbol substitution (Z) | -3.24 | -3.0 | -2.4* |
| **Working Memory** |  |  |  |
| Digit span forward (Z) | -2.41 | -2.4 | -0.5** |
| **Visuospatial construction** |  |  |  |
| Rey-O copy (T) | <0 | <0 | <0 |
| **Visual memory** |  |  |  |
| Rey-O immediate recall (T) | 22 | 28 | 26 |
| Rey-O delayed recall 15 mins (T) | <20 | 26* | 27* |
| **Verbal memory** |  |  |  |
| HVLT-Trials 1-3 (T) | <20 | <20 | 32* |
| HVLT-Delay 20 mins (T) | 25 | <20^ | <20^ |
| HVLT-Retention (T) | 57 | 57 | 56 |
| HVLT-Recognition (Total/12) | 12 | 12 | N/A |
| HVLT-Discrimination (T) | 25 | ≤20^ | N/A |
| **Psychological Functioning** |  |  |  |
| GDS (total score) | 2 | 2 | 2 |
| NPI-Q (total score) | 8 | N/A | N/A |

| **PPA05 (LIFG)** | **Baseline** | **Post** | **Follow up** |
| --- | --- | --- | --- |
| BNT-2 (T) | 45 | 45 | 45 |
| PPT (Total/52) | 50 | 50 | 51 |
| COWAT “FAS” (T) | 25 | 40** | 43 † |
| Category fluency “Animals” (T) | 49 | 49 | 45 |
| **Executive Function** |  |  |  |
| Trails A/ B (T) | 39/39 | 39/43 | 35/43 |
| Digit span backward (Z) | -2.3 | 0.2** | -1.5 |
| Digit symbol substitution (Z) | -1.5 | -0.8 | -0.7 |
| **Working Memory** |  |  |  |
| Digit span forward (Z) | -1.6 | -2.5 | -1.6 |
| **Visuospatial construction** |  |  |  |
| Rey-O copy (T) | 47 | 58* | 53 |
| **Visual memory** |  |  |  |
| Rey-O immediate recall (T) | 51 | 74** | 51 |
| Rey-O delayed recall 15 mins (T) | 51 | 65* | 48 |
| **Verbal memory** |  |  |  |
| HVLT-Trials 1-3 (T) | 39 | 54** | 41 |
| HVLT-Delay 20 mins (T) | 48 | 52 | 48 |
| HVLT-Retention (T) | 44 | 62* | 50 |
| HVLT-Recognition(Total/12) | 11 | 12 | 12 |
| HVLT-Discrimination (T) | 53 | 56 | 50 |
| **Psychological Functioning** |  |  |  |
| GDS (total score) | 6 | 3 | 3 |
| NPI-Q (total score) | 3 | 0 | N/A |

| **PPA07 (LIFG)** | **Baseline** | **Post** | **Follow up** |
| --- | --- | --- | --- |
| BNT-2 (T) | 45 | 42 | 49 |
| PPT (Total/52) | 51 | 49 | 51 |
| COWAT “FAS” (T) | 46 | 54 | 40 |
| Category fluency “Animals” (T) | 32 | 49** | 45† |
| **Executive Function** |  |  |  |
| Trails A/ B (T) | 33/53 | 37/44 | 50**/53 |
| Digit span backward (Z) | -1.0 | -0.1 | -0.1 |
| Digit symbol substitution (Z) | -1.2 | 0.4* | -0.1† |
| **Working Memory** |  |  |  |
| Digit span forward (Z) | -0.9 | -0.1 | 0.0 |
| **Visuospatial construction** |  |  |  |
| Rey-O copy (T) | 58 | 55 | 58 |
| **Visual memory** |  |  |  |
| Rey-O immediate recall (T) | 79 | 79 | >80 |
| Rey-O delayed recall 15 mins (T) | 68 | 73 | 75 |
| **Verbal memory** |  |  |  |
| HVLT-Trials 1-3 (T) | 55 | 48 | 52 |
| HVLT-Delay 20 mins (T) | 53 | 52 | 52 |
| HVLT-Retention (T) | 48 | 62* | 56 |
| HVLT-Recognition (Total/12) | 12 | 12 | 12 |
| HVLT-Discrimination (T) | 48 | 51 | 51 |
| **Psychological Functioning** |  |  |  |
| GDS (total score) | 0 | 2 | 4 |
| NPI-Q (total score) | 3 | N/A | N/A |

| **PPA08 (LIFG)** | **Baseline** | **Post** | **Follow up** |
| --- | --- | --- | --- |
| BNT-2 (T) | 41 | 45 | 41 |
| PPT (Total/52) | 50 | 51 | 51 |
| COWAT “FAS” (T) | 32 | 39 | 28 |
| Category fluency “Animals” (T) | 27 | 44** | 40** |
| **Executive Function** |  |  |  |
| Trails A/ B (T) | 55/47 | 50/51 | 50/51 |
| Digit span backward (Z) | -1.6 | 0.1* | -0.8 |
| Digit symbol substitution (Z) | 0.3 | 0.9 | 0.8 |
| **Working Memory** |  |  |  |
| Digit span forward (Z) | -1.8 | -2.7 | -1.8 |
| **Visuospatial construction** |  |  |  |
| Rey-O copy (T) | 17 | 34* | 38* |
| **Visual memory** |  |  |  |
| Rey-O immediate recall (T) | 47 | 75** | 61* |
| Rey-O delayed recall 15 mins (T) | 47 | 71** | 64* |
| **Verbal memory** |  |  |  |
| HVLT-Trials 1-3 (T) | 44 | 52* | 62* |
| HVLT-Delay 20 mins (T) | 53 | 56 | 63* |
| HVLT-Retention (T) | 50 | 56 | 56 |
| HVLT-Recognition (Total/12) | 12 | 11 | 12 |
| HVLT-Discrimination (T) | 59 | 50 | 56 |
| **Psychological Functioning** |  |  |  |
| GDS (total score) | 2 | 1 | 5 |
| NPI-Q (total score) | 0 | N/A | 2 |

| **PPA09 (pre-SMA)** | **Baseline** | **Post** | **Follow up** |
| --- | --- | --- | --- |
| BNT-2 (T) | 51 | 55 | 48 |
| PPT (Total/52) | 50 | 52 | 52 |
| COWAT “FAS” (T) | 17 | 24 | 24 |
| Category fluency “Animals” (T) | 19 | 27 | 40** |
| **Executive Function** |  |  |  |
| Trails A/ B (T) | 27/DC | 31/DC | 31/27* |
| Digit span backward (Z) | -0.8 | -1.6 | -0.8 |
| Digit symbol substitution (Z) | -3.0 | -2.5 | -3.1 |
| **Working Memory** |  |  |  |
| Digit span forward (Z) | 0.2 | -0.7 | -1.7^ |
| **Visuospatial construction** |  |  |  |
| Rey-O copy (T) | 40 | 23^ | 19^ |
| **Visual memory** |  |  |  |
| Rey-O immediate recall (T) | 40 | 32 | 42 |
| Rey-O delayed recall 15 mins (T) | 31 | 35 | 32 |
| **Verbal memory** |  |  |  |
| HVLT-Trials 1-3 (T) | 38 | 38 | 40 |
| HVLT-Delay 20 mins (T) | 38 | 38 | 42 |
| HVLT-Retention (T) | 43 | 43 | 56* |
| HVLT-Recognition (Total/12) | 10 | 10 | 10 |
| HVLT-Discrimination (T) | 43 | 45 | 45 |
| **Psychological Functioning** |  |  |  |
| GDS (total score) | 3 | 4 | 5 |
| NPI-Q (total score) | 8 | N/A | 12 |

| **PPA11 (LIFG)** | **Baseline** | **Post** | **Follow up** |
| --- | --- | --- | --- |
| BNT-2 (T) | 16 | 23 | 16 |
| PPT (Total/52) | 42 | 44 | 44 |
| COWAT “FAS” (T) | 15 | 19 | 33* |
| Category fluency “Animals” (T) | 21 | 25 | 17 |
| **Executive Function** |  |  |  |
| Trails A/ B (T) | 18/NA | 27/NA | 18/NA |
| Digit span backward (Z) | -2.4 | -1.5 | -1.5 |
| Digit symbol substitution (Z) | -2.1 | -1.0* | -2.0 |
| **Working Memory** |  |  |  |
| Digit span forward (Z) | -2.6 | -2.6 | -2.6 |
| **Visuospatial construction** |  |  |  |
| Rey-O copy (T) | 1 | 0 | <0 |
| **Visual memory** |  |  |  |
| Rey-O immediate recall (T) | 7 | 29** | 34* |
| Rey-O delayed recall 15 mins (T) | <20 | <20 | 37** |
| **Verbal memory** |  |  |  |
| HVLT-Trials 1-3 (T) | 21 | 27 | 29 |
| HVLT-Delay 20 mins (T) | 27 | 31 | 27 |
| HVLT-Retention (T) | 56 | 56 | 28^ |
| HVLT-Recognition (Total/12) | 6 | 8 | 10 |
| HVLT-Discrimination (T) | ≤20 | 29* | 35* |
| **Psychological Functioning** |  |  |  |
| GDS (total score) | 1 | 1 | N/A |
| NPI-Q (total score) | 5 | 1 | N/A |

**Scores which improve from impaired to normal range or by 2 standard deviations (Jacobson et al., 1984), *Scores which improved by 1 standard deviation or improved from below the lowest possible score to a measurable score. †Clinically significant maintenance. ^clinically significant decline (by at least 1 standard deviation or dropping to below the lowest possible score).

**Supplementary Table 3: EEG task performance and ERP results**

| **SC task** | | | | | | | |
| --- | --- | --- | --- | --- | --- | --- | --- |
|  | **Go RT** | **Go Acc** | **Nogo Acc** | **N2 latency** | **N2 amplitude** | **P3 latency** | **P3 amplitude** |
| **NC (n = 7)** | | | | | | | |
|  | 375 (71) | 94.6 (9.9) | 88.5 (14.9) | 293 (48.9) | 0.49 (1.19) | 456 (60.1) | 0.77 (0.96) |
| **PPA-all (n = 8)** | | | | | | | |
| **baseline** | 539 (150) | 94.1 (9.3) | 84.0 (14.9) | 264 (48.9) | 0.12 (1.20) | 530 (60.3) | 1.24 (0.96) |
| **post*** | 566.3 (174.1) | 95.9 (9.6) | 91.9 (10.8) | 269.2 (33.6) | -0.12 (1.26) | 518.9 (48.6) | 1.26 (0.89) |
| **PPA LIFG (n = 4)** | | | | | | | |
| **baseline** | 477 (258.2) | 93.4 (15.4) | 85.2 (22.8) | 281 (76.1) | -0.50 (1.50) | 516 (92.5) | 1.69 (1.51) |
| **post*** | 483 (249) | 93.3 (15.8) | 96.1 (24.2) | 272 (83.6) | -0.88 (1.62) | 505 (101.1) | 1.15 (1.59) |
| **PPA pre-SMA (n = 4)** | | | | | | | |
| **baseline** | 600.8 (161.9) | 94.8 (6.0) | 82.7 (11.6) | 247 (73.7) | 0.25 (0.90) | 526.9 (72.9) | 0.79 (1.02) |
| **post** | 600.8 (169.5) | 99.2 (0.6) | 89.6 (14.5) | 267 (39.1) | 0.40 (1.26) | 530.8 (43.8) | 1.11 (1.15) |

| **OA task** | | | | | | | |
| --- | --- | --- | --- | --- | --- | --- | --- |
|  | **Go RT** | **Go Acc** | **Nogo Acc** | **N2 latency** | **N2 amplitude** | **P3 latency** | **P3 amplitude** |
| **NC (n = 7)** | | | | | | | |
|  | 475 (107) | 88.5 (9.9) | 82.9 (14.9) | 256 (48.9) | 0.26 (1.19) | 530 (60.1) | 0.61 (0.96) |
| **PPA-all (n = 8)** | | | | | | | |
| **baseline** | 648 (150) | 88.5 (9.3) | 70.3 (14.9) | 288 (48.9) | -0.48 (1.20) | 545 (60.3) | 1.11 (0.96) |
| **post*** | 631.0 (174.4) | 92.7 (10.9) | 76.8 (11.1) | 282.6 (57.5) | -0.50 (1.50) | 524.3 (83.6) | 0.26 (1.07) |
| **PPA LIFG (n = 4)** | | | | | | | |
| **baseline** | 575 (258.2) | 90.9 (15.5) | 73.8 (22.8) | 294 (76.1) | -0.51 (1.50) | 555 (92.5) | 1.09 (1.51) |
| **post*** | 548 (249) | 91.5 (15.8) | 76.9 (24.2) | 307 (83.6) | -1.28 (1.62) | 474 (101.1) | -0.79 (1.59) |
| **PPA pre-SMA (n = 4)** | | | | | | | |
| **baseline** | 721.7 (245.7) | 86.1 (17.4) | 66.9 (31.0) | 282.4 (70.7) | -0.46 (0.49) | 526.6 (86.9) | 1.13 (0.91) |
| **post** | 665.4 (186.6) | 95.0 (4.2) | 77.5 (10.4) | 264.0 (71.9) | 0.06 (0.79) | 563.3 (19.7) | 0.81 (1.00) |

**post-treatment data missing for PPA08 assigned to LIFG group*

**References for Supplementary materials:**

Benedict, R. H. B., Schretlen, D., Groninger, L., & Brandt, J. (1991). Hopkins Verbal Learning Test--Revised (HVLT-R) (Database record). APA PsycTests.

Chiang, H. S., Motes, M., Afkhami-Rohani, B., Adhikari, A., LoBue, C., Kraut, M., Cullum, C. M., & Hart, J., Jr (2024). Verbal retrieval deficits due to traumatic brain injury are associated with changes in event related potentials during a Go-NoGo task. *Clinical neurophysiology : official journal of the International Federation of Clinical Neurophysiology*, *163*, 1–13. <https://doi.org/10.1016/j.clinph.2024.04.002>

Cumming, G. (2013). *Understanding the new statistics: Effect sizes, confidence intervals, and meta-analysis*. Routledge. https://doi.org/10.4324/9780203807002

Dalton, S. G., AL Harbi, M., Berube, S., & Hubbard, H. I. (2024). Development of main concept and core lexicon checklists for the original and modern Cookie Theft stimuli. *Aphasiology*, 1-25. https://doi.org/10.1080/02687038.2024.2340794

Delorme, A., & Makeig, S. (2004). EEGLAB: An open source toolbox for analysis of single-trial EEG dynamics including independent component analysis. Journal of Neuroscience Methods, 134, 9–21.

Jacobson, N. S., Follette, W. C., & Revenstorf, D. (1984). Psychotherapy outcome research: Methods for reporting variability and evaluating clinical significance. *Behavior therapy*, *15*(4), 336-352. https://doi.org/10.1016/S0005-7894(84)80002-7

Kaufer D, Cummings J, Ketchel P, Smith V, MacMillan A, Shelley T, et al. Validation of the NPI-Q, a brief clinical form of the Neuropsychiatric Inventory. J Neuropsychiatry Clin Neurosci. 2000;12:233–239.

Kim, H., Kintz, S., & Wright, H. H. (2021). Development of a measure of function word use in narrative discourse: Core lexicon analysis in aphasia. *International journal of language & communication disorders*, *56*(1), 6-19. https://doi.org/10.1111/1460-6984.12567

Kong, A. P. H. (2009). The use of main concept analysis to measure discourse production in Cantonese-speaking persons with aphasia: A preliminary report. *Journal of Communication Disorders*, *42*(6), 442-464. https://doi.org/10.1016/j.jcomdis.2009.06.002

Partington JE, Leiter GR. Partington’s pathways test. Psychol Serv Cebter J 1949;1:11–20.

Pion-Tonachini, L., Kreutz-Delgado, K., & Makeig, S. (2019). ICLabel: An automated electroencephalographic independent component classifier, dataset, and website. NeuroImage, 198, 181–197.

Rey, A., & Osterrieth, P. A. (1941). Rey-Osterrieth Complex Figure Copying Test (Database record). APA PsycTests.

Richardson, J. D., & Dalton, S. G. (2016). Main concepts for three different discourse tasks in a large non-clinical sample. *Aphasiology*, *30*(1), 45-73. https://doi.org/10.1080/02687038.2015.1057891

Richardson, J. D., & Dalton, S. G. H. (2020). Main concepts for two picture description tasks: an addition to Richardson and Dalton, 2016. *Aphasiology*, *34*(1), 119-136. <https://doi.org/10.1080/02687038.2018.1561417>

Sheikh, J. I., & Yesavage, J. A. (1986). Geriatric Depression Scale (GDS): Recent evidence and development of a shorter version. Clinical Gerontologist: The Journal of Aging and Mental Health, 5(1-2), 165–173

Wechsler D. Wechsler Adult Intelligence Scale—Fourth Edition. Bloomington, MN: PsychCorp, an imprint of Pearson Clinical Assessment; 2008.
